# Clinical applications of palmitoylethanolamide in pain management: protocol for a scoping review

**DOI:** 10.1186/s13643-018-0934-z

**Published:** 2019-01-08

**Authors:** Maria Beatrice Passavanti, Aniello Alfieri, Maria Caterina Pace, Vincenzo Pota, Pasquale Sansone, Giacomo Piccinno, Manlio Barbarisi, Caterina Aurilio, Marco Fiore

**Affiliations:** 1 Department of Women, Child and General and Specialized Surgery, University of Campania “Luigi Vanvitelli”, Piazza L. Miraglia, 2, 80138 Naples, Italy; 2 Department of Medical, Surgical, Neurological, Metabolic and Aging Sciences, University of Campania “Luigi Vanvitelli”, Piazza L. Miraglia, 2, 80138 Naples, Italy

**Keywords:** Palmitoylethanolamide, Systematic review, PEA, Pain, Pain management, Endocannabinoids, Ethanolamines, Humans, Palmitic acids, Palmidrol

## Abstract

**Background:**

Palmitoylethanolamide (PEA) belong to endocannabinoid family, a group of fatty acid amides. PEA has been proven to have analgesic and anti-inflammatory activity and has been used in several controlled studies focused on the management of chronic pain among adult patients with different underlying clinical conditions.

**Methods/design:**

A literature search will be performed using PubMed, EMBASE, and the Cochrane Central Register of Controlled Trials (CENTRAL). The population will be patients who have chronic pain, the intervention will be the administration of PEA alone or in combination with other drugs for the pain management; the comparison will be the standard therapy in accordance with the current guidelines for the treatment of pain. The Outcomes will be the reduction of pain not restricted to specific scales laying out the pain outcome data described in the included studies.

**Discussion:**

This scoping review aims to describe the clinical applications of the PEA in chronic pain management and its outcome.

**Scoping review registration:**

Open Science Framework https://osf.io/74tmx/.

## Background

Palmitoylethanolamide (PEA) is an endogenous fatty acid amide, an analog of the endocannabinoid anandamide (AEA), that belongs to the family of N-acylethanolamines (NAE) [1]. NAEs are released from cells in response to noxious stimuli. As all NAEs, also the PEA has a local effect, and its tissue levels are closely regulated through the balance of production and degradation activity. Two intracellular amidases, expressed in the inflammatory cells, have been involved in lipid amide degradation: fatty-acid amide hydrolase (FAAH) and N-acylethanolamine hydrolyzing acid amidase (NAAA) [2].

The effects of the PEA are due to its interaction with several pathways: at first, it reduces, via the peroxisome proliferator-activated receptor alpha (PPARα), the recruitment and activation of mast cells at sites of nerve injury and the release of pro-inflammatory mediators from these cells [3, 4]; secondly, it inhibits the microglia activation and the recruitment of mast cells into spinal cord after peripheral nerve injury, as well as following spinal neuroinflammation or spinal cord injury [5, 6]. In the beginning, PEA was also supposed to be an agonist of the cannabinoid type II receptor (CB2) [7]; subsequently, in their research, Sugiura et al. have demonstrated that PEA has just a very low affinity for this receptor [8], clarifying why CB2 antagonists do not inhibit some of its anti-inflammatory effects [9]. Anyhow, PEA indirectly activates CB2 and the cannabinoid receptor type 1 (CB1) [10], down-modulating fatty acid amide hydrolase (FAAH), the enzyme responsible of the degradation of the anandamide (AEA), a CB1 agonist [11].

Several studies focused on the use of PEA in a multitude of chronic pain conditions. For example, it can have a beneficial effect like adjuvant for the treatment of the low back pain [12] or it was used alone for chronic pain management in critically ill older patients, where the use of traditional analgesics can lead to high risk of adverse effect [13]. Encouraging results have been shown in the treatment of non-surgical radiculopathies with an ultra-micronized formulation of PEA [14] and the combination therapy with alpha-lipoic acid to reduce chronic prostatitis/chronic pelvic pain syndrome [15].

### Importance of this review

Although pharmacological pain therapy offers several alternatives, pain management remains often unsatisfactory. In order to reinforce the therapeutic solutions, the use of the PEA for the treatment of chronic or inflammatory pain may be a valid strategy. To our knowledge, this is the first scoping review that summarizes the literature findings on the use of PEA in chronic pain management.

## Methods/design

### Research questions

This review is designed to answer the following research question:

#### What are the current clinical applications of PEA in the management of chronic pain?

We will prepare this scoping review according to the Preferred Reporting Items for Systematic Reviews and Meta-Analysis Extension for Scoping Reviews (PRISMA-ScR) [16].

### Searching

A literature search will be performed using several computer-assisted databases, including PubMed, EMBASE, and the Cochrane Central Register of Controlled Trials (CENTRAL). To the results, we will add the publications cited in articles obtained by primary research, previous reviews, or books to identify additional eligible studies.

The search strategy and the search string will be formulated following the PICO method.

The Population will be patients who have chronic pain, and the Intervention will be the administration of PEA alone or in combination with other drugs for the treatment of pain. The Comparator will be the standard therapy in accordance with the current guidelines for the treatment of pain. The Outcomes will be pain reduction measured with any type of pain assessment scale.

Computer searches will be performed using the following search string: “palmitoylethanolamide” AND “pain.”

The reference list of the retrieved articles will be used to find relevant studies that will be not allocated through the searching procedure. We will not restrict the search with any filter. Duplicates will be removed after the literature search, and two reviewers (AA and GP) will independently conduct a two-stage screening reading the titles and abstracts identified in the search strategy detailed above. Each title will be screened using a screening guide. Titles will be retained if they appear to meet the inclusion criteria or if it is uncertain if they do (Table 1).

**Table 1:** Eligibility criteria

	Inclusion	Exclusion
Study design	Primary studies of any design that includes a control group	Systematic reviews
Population	Patients who have chronic pain	n/a
Intervention/exposure	Administration of PEA alone or in combination	n/a
Outcomes	Pain reduction assessed with all approaches available for assessing pain intensity	Anything other than the selected outcomes
Language	English	Anything other than English
Publication status	Published in peer review journals, full-length articles	Published in not peer-review journals, unpublished works as a full-text, abstract, conference meetings
Others	All study dates, length of follow-up, setting	n/a

### Eligibility criteria

To be included in the review, studies will need to have a control group, in which patients with chronic pain are explicitly treated with PEA, with no restrictions on publication year. We will exclude unpublished works as a full-text, abstract, conference meetings, studies published in not peer-review journals, uncontrolled studies as case series or case reports, reviews, and studies published not in English.

Papers will be excluded if they do not fit into the conceptual framework of the study, focused on chronic pain management.

### Primary abstract screening

Initially, the articles will be selected by the authors assessing titles and abstracts to identify potentially eligible studies; then, the full-text of the eligible studies will be reviewed by the authors to exclude irrelevant studies or methodologies not being a useful motivation for future analysis.

### Methods for data extraction

The reviewers will record key information from included articles in a Microsoft Excel data extraction form designed a priori. Two reviewers (AA and GP) will independently extract data to minimize errors. Each study will be extracted with the following information: title, year of publication, first author, the country where the study was conducted, type of study, lying chronic disease for which the PEA was used, and outcome.

### Strategy for data synthesis

The number of studies identified and selected at each stage of the scoping review and the reasons for exclusion will be presented in a PRISMA flow diagram. Results will be summarized in table form (Table 2) and discussed deeper in narrative form to address the research questions. Results will be grouped conceptually, by general study details, study characteristics, participants, interventions/exposures/comparators, instruments used in goal-setting, outcomes, and results. This review will present summaries of these categories, including quantitative measurements of associations (mean differences for scores by validated questionnaires, risk ratios, or odds ratios for dichotomous outcomes), if applicable. Additional groups may be identified during the extraction of results.

**Table 2:** Planned variables to be extracted in the scoping review

General study details	Study ID number, lead author, title, journal, year of publication, type of publication, information source
Study characteristics	Study design, study duration, pilot/feasibility study (y/n), number of study arms, covariates (definition and measurement methods)
Participants	1. Total number, setting, inclusion and exclusion criteria 2. Participant characteristics at baseline: for each study, average age (years, mean and standard deviation [SD]), sex (%), country, and time since diagnosis (or time since treatment, if treatment was completed)
Interventions/exposures and comparators	1. Total number of intervention/exposure and comparison groups and number of participants in each group 2. For each intervention/exposure and comparison group: intervention/exposure/comparison, duration of intervention/exposure, who and how assessed, and results of assessment
Outcomes	The approach used in the study for assessing pain intensity including categorical scales (e.g., mild, moderate, severe), numerical rating scales (NRS), visual analog scales (VAS), and well-validated verbal scales (eg, the Descriptor Differential Scale).
Results	For each quantitative outcome: sample size, number of missing participants, reasons for loss to follow up, summary data for each group (2 × 2 table for dichotomous data, means and SDs for continuous data), estimate of effect for the difference between groups (or change in baseline and final scores for single-arm studies), confidence intervals, and *p* value

Authors of papers will be contacted to request missing or additional data for clarification, where required. We will report the results of critical appraisal in narrative form and in a table.

The final protocol was registered prospectively with the Open Science Framework on 12 December 2018 (https://osf.io/74tmx/).

## Discussion

This protocol is for a scoping review that is planned and not started. This scoping review aims to describe the clinical applications of the PEA in pain management of different chronic diseases and its outcome.

## Abbreviations

AEA: Anandamide
CB1: Cannabinoid type I receptor
CB2: Cannabinoid type II receptor
CENTRAL: Cochrane Central Register of Controlled Trials
FAAH: Fatty-acid amide hydrolase
NAAA: N-acylethanolamine hydrolyzing acid amidase
NAE: N-acylethanolamines
PEA: Palmitoylethanolamide
PPARα: Peroxisome proliferator-activated receptor alpha
PRISMA-P: Preferred Reporting Items for Systematic Reviews and Meta-Analysis Protocols
VAS Pain: Visual Analog Scale for Pain
